# MTT-Based Cytotoxicity Assessment of Chitosan- and Octenisept^®^-Biomodified Mineral Trioxide Aggregate in Human Periodontal Ligament Fibroblasts

**DOI:** 10.3390/biomimetics11080528

**Published:** 2026-07-29

**Authors:** Ilgın İlgenli, Esra İsmailoğlu, Pelin İlhan

**Affiliations:** 1 Department of Endodontics, Faculty of Dentistry, Ege University, 35040 Izmir, Turkey; 2 Department of Biotechnology, Graduate School of Natural and Applied Sciences, Ege University, 35100 Izmir, Turkey; 3 PA Biotechnology Industry Trade Inc., Ege Technopark Technology Development Zone, 35100 Izmir, Turkey

**Keywords:** mineral trioxide aggregate, chitosan, Octenisept^®^, biomodification, cytotoxicity, MTT assay, human periodontal ligament fibroblasts, calcium silicate-based materials, endodontic repair materials

## Abstract

Calcium silicate-based repair materials are widely used in endodontics, and bioinspired polymeric biomodification may influence their cellular response. However, the cytotoxicity profile of mineral trioxide aggregate (MTA) modified with Octenisept^®^ or with an acetic acid-based chitosan vehicle has not been specifically evaluated in a controlled design comparing chitosan-modified MTA groups under parallel MTA extract and direct solution exposure conditions, although biomodifier effects may differ when tested alone and when incorporated into an MTA matrix. This in vitro study evaluated the MTT-based cytotoxicity profile of MTA subjected to bioinspired chitosan-based biomodification in human periodontal ligament fibroblasts (hPDLFs), using Octenisept^®^ as an antiseptic comparator biomodifier and 1% acetic acid as the vehicle control. Extracts of unmodified-MTA, MTA + 1% acetic acid, MTA + 1% chitosan, MTA + 3% chitosan and MTA + Octenisept^®^ were tested at 100%, 50% and 25% concentrations. The corresponding solutions were tested under direct exposure at 100%, 50%, 25% and 12.5%. Cell viability was assessed after 24 and 72 h using the MTT assay, and values below 70% were considered cytotoxic according to ISO 10993-5. Direct solution exposure produced stronger cytotoxicity, particularly for Octenisept^®^. Among the MTA extract groups, only MTA + 1% chitosan maintained non-cytotoxic cell viability at 72 h under undiluted extract conditions.

## 1. Introduction

Calcium silicate-based materials are widely used in endodontic repair procedures because of their biocompatibility, bioactive behaviour, and ability to support hard tissue formation [1,2]. Among these materials, mineral trioxide aggregate (MTA) is a bioceramic-based repair material commonly used in clinical procedures such as pulp capping, perforation repair, apical barrier formation, and root-end filling [3,4]. The biological behaviour of MTA is largely related to its hydration reaction in a moist environment. During this process, calcium silicate hydrate structures and calcium hydroxide are formed, the pH of the environment increases, and calcium ion release occurs [5,6]. This alkaline and calcium-rich environment may support mineralisation and tissue repair; however, it may also influence the early cellular response to the material [6,7].

Endodontic repair materials are often placed close to both microbially contaminated areas and periradicular tissues, particularly during perforation repair, apical barrier formation, and root-end filling procedures. Therefore, biomodification strategies aimed at improving the antimicrobial potential of MTA represent a clinically relevant research area. However, antimicrobial or biopolymeric agents added to MTA should be evaluated not only for their effects on microorganisms, but also for their biological effects on cells associated with periradicular tissues [3,8]. Thus, antimicrobial potential and periapical cell response should be considered together when evaluating MTA biomodifications.

Although MTA is generally regarded as a biocompatible material, the cellular response may vary depending on the setting state of the material, extract concentration, and exposure time [8,9,10]. Previous studies have shown that freshly mixed or early-stage material extracts may affect cell viability more markedly, whereas biological tolerance may improve as the setting process progresses [9,10]. Therefore, when evaluating MTA biomodifications, different extract concentrations and exposure periods should be considered to better understand the cellular response.

Persistent microorganisms in endodontic infections are important factors that may adversely affect treatment success [11,12,13]. Although the antimicrobial effect of MTA is mainly associated with its highly alkaline pH, this effect may vary according to the microorganism, contact time, and experimental conditions [6,7,14]. This has led to the development of additive-based or biomodification approaches that may improve the antimicrobial properties of MTA. However, such modifications may also affect the cellular compatibility of MTA [15,16,17].

Octenidine dihydrochloride is a broad-spectrum cationic antiseptic that exerts its antimicrobial effect by interacting electrostatically with negatively charged components of the microbial cell membrane, thereby disrupting membrane integrity [18,19,20]. Octenisept^®^ is a commercial antiseptic solution containing 0.1% octenidine dihydrochloride and 2% phenoxyethanol. In the endodontic literature, octenidine has been reported to be effective against resistant microorganisms such as Enterococcus faecalis and Candida albicans, and to show antimicrobial activity comparable to sodium hypochlorite (NaOCl) or chlorhexidine gluconate (CHX) under certain conditions [21,22,23]. However, the effects of alternative antiseptics such as Octenisept^®^ on eukaryotic cells also need to be evaluated [24,25,26].

Chitosan is a natural, cationic, and biodegradable biopolymer obtained by the deacetylation of chitin. It has been investigated in biomedical applications because of its biocompatibility, antimicrobial activity, wound-healing properties, and potential use in regenerative procedures [27,28]. From a biomimetic perspective, chitosan is relevant not only because of its natural origin, but also because its polysaccharide backbone, cationic amino groups, and film-forming capacity provide structural and functional features that resemble selected aspects of extracellular matrix-associated biopolymers. These properties make chitosan a bioinspired candidate for modifying calcium silicate-based repair materials, particularly when the aim is to combine antimicrobial functionality with a cell-compatible material interface. The antimicrobial effect of chitosan is attributed to electrostatic interactions between its protonatable amino groups and negatively charged components on microbial cell surfaces [29,30,31]. However, the biological behaviour of chitosan is affected by several variables, including concentration, degree of deacetylation, solubility, viscosity, and pH [31,32,33]. Because chitosan has limited solubility at neutral pH, it is commonly dissolved in weak acidic vehicles such as acetic acid in experimental applications [31,34]. Therefore, in MTA systems containing chitosan, the possible effect of the acetic acid vehicle on the cellular response should be assessed using a separate control group.

Periodontal ligament fibroblasts play an important role in root-surrounding tissue homeostasis, extracellular matrix metabolism, wound healing, and periodontal regeneration [35,36]. Since MTA and similar endodontic materials may contact periradicular tissues in clinical situations such as root-end applications, perforation repair, or apical extrusion, human periodontal ligament fibroblasts (hPDLFs) provide a biologically relevant cell model for evaluating the cellular response to these materials [37,38].

Although the cytotoxicity profile of calcium silicate-based materials has been investigated, studies comparatively evaluating the time- and concentration-dependent cytotoxicity of biomodified MTA formulations remain limited. In previous studies, the cellular response was often assessed at a single time point, the vehicle effect was not examined as a separate control, or the effect of antimicrobial additives on cellular compatibility was not compared with unmodified MTA [25,39]. These factors make it difficult to determine whether the observed cellular response is related to the base material, the biomodifying agent, or the vehicle. The unique contribution of the present study is the controlled comparative design, in which Octenisept^®^-modified MTA, chitosan-modified MTA, unmodified MTA, and a separate acetic acid vehicle control were evaluated in parallel with the corresponding direct solution exposure groups. This design allowed the effects of the MTA matrix, biomodifier type, vehicle system, extract concentration, and exposure time to be considered together. To the best of our knowledge, the comparative MTT-based cellular response of MTA modified with Octenisept^®^ or with an acetic acid-based chitosan vehicle has not been specifically evaluated using this parallel combined extract/direct solution exposure approach in hPDLFs. This represents an important gap because the biological behaviour of a biomodifier as an isolated solution may differ from its behaviour after incorporation into an MTA matrix. Therefore, the present study was designed to evaluate the MTT-based cytotoxicity profile of MTA subjected to bioinspired chitosan-based biomodification, using Octenisept^®^ as an antiseptic comparator biomodifier and 1% acetic acid as the vehicle control, in hPDLF cells. For this purpose, extracts of unmodified MTA, MTA + 1% acetic acid, MTA + 1% chitosan, MTA + 3% chitosan, and MTA + Octenisept^®^ were evaluated at 100%, 50%, and 25% concentrations. The corresponding solutions were also tested without being mixed with MTA at 100%, 50%, 25%, and 12.5% concentrations. MTT-based relative cell viability was assessed after 24 and 72 h of exposure. This controlled design enabled a comparison of the selected biomodifiers both within the MTA matrix and under direct solution exposure conditions, using unmodified MTA and the acetic acid vehicle as comparators. This approach was intended to provide controlled preliminary cytotoxicity data for selecting candidate MTA biomodifications for further biological, antimicrobial, and physicochemical evaluation. The null hypothesis was that MTA biomodifications and the corresponding solutions would not affect MTT-based hPDLF cell viability at the tested concentrations after 24 and 72 h of exposure.

## 2. Materials and Methods

This was an in vitro study using hPDLFs that were obtained from previously established primary hPDLFs cell stocks isolated with the approval of the Ege University Faculty of Medicine Ethics Committee (Approval No: 02-5/14) and supplied by PA Biyoteknoloji AŞ (Izmir, Turkey). No new human participants were recruited for the present study, and no identifiable personal data were used.

### 2.1. Study Design and Experimental Groups

In this in vitro study, the MTT-based cellular response of hPDLF cells to MTA prepared with different biomodifications was evaluated. The experimental design included three main categories: control groups, MTA-containing extract groups, and direct solution exposure groups used to evaluate the effect of the solutions alone. A total of 11 groups were established. The control groups consisted of:

G1: negative control, untreated cells with culture medium;

G2: positive control, 10% dimethyl sulfoxide (DMSO), used to confirm the cytotoxic response.

The MTA-containing extract groups were as follows:

G3: unmodified MTA, used as the reference material;

G4: MTA + 1% acetic acid;

G5: MTA + 1% chitosan prepared in 1% acetic acid;

G6: MTA + 3% chitosan prepared in 1% acetic acid;

G7: MTA + Octenisept^®^.

The direct solution exposure groups were as follows:

G8: 1% acetic acid;

G9: 1% chitosan + 1% acetic acid;

G10: 3% chitosan + 1% acetic acid;

G11: Octenisept^®^.

Disc specimens were prepared only for the MTA-containing extract groups. In the solution groups, the direct cytotoxic effects of the corresponding solutions on hPDLF cells were evaluated. This controlled design was used to distinguish the cellular effects of the biomodifying agents incorporated into the MTA matrix from their effects under direct solution exposure. The overall experimental design and grouping scheme are summarized in Figure S1.

### 2.2. Cell Culture

Cells were cultured in Dulbecco’s modified Eagle’s medium (DMEM)-high glucose supplemented with 10% fetal bovine serum (FBS), 1% L-glutamine and 0.1% gentamicin. They were maintained at 37 °C in a humidified incubator containing 5% CO_2_. All cell culture procedures were performed under aseptic conditions in a Class II biosafety cabinet. hPDLFs at passage 8 were used for all experiments. Frozen cells were removed from −86 °C storage and thawed in a 37 °C water bath. The cell suspension was transferred into 15 mL tubes, mixed with 4 mL of fresh culture medium and centrifuged at 1000 rpm for 5 min at +4 °C. After centrifugation, the supernatant was removed, and the cells were resuspended in fresh medium and transferred into T75 culture flasks. When the cells reached approximately 80% confluence, they were passaged. The culture medium was removed, and the cells were washed with Ca^2+^- and Mg^2+^-free phosphate-buffered saline (PBS). Then, 0.05% trypsin/EDTA was added, and the cells were incubated at 37 °C for approximately 5 min. After cell detachment, trypsin was neutralized by adding at least three volumes of culture medium. The cell suspension was transferred into 15 mL tubes and centrifuged at 1000 rpm for 5 min at +4 °C. The supernatant was removed, and the cells were resuspended in fresh medium and passaged at a 1:3 ratio. Before the experiment, the cells were detached by trypsinization and counted using trypan blue staining. Cells were seeded in 96-well plates at a density of 1 × 10^5^ cells/mL and incubated for 24 h to allow cell attachment before extract and solution application.

### 2.3. Preparation of Materials

The minimum required sample size was five per experimental condition, assuming an alpha error probability of 0.05 and a statistical power of 0.80.

Biofactor MTA (Imicryl Dental, Konya, Turkey) is supplied as a powder-liquid system. Chitosan CP200–300 (ZAG Kimya, İstanbul, Turkey) was used as the chitosan source. Because chitosan has limited solubility under neutral pH conditions, it was dissolved in an acidic medium for experimental use. For this purpose, 1% acetic acid (Izmir Teknik Kimya, İzmir, Turkey) was used as the vehicle. Chitosan becomes soluble through protonation in acidic media [40]. Octenisept^®^ (Schülke & Mayr GmbH, Norderstedt, Germany) was used in its commercial form. A summary of the materials and solutions used in the study, including supplier, composition and catalogue/lot information when available was given in Table 1.

All glassware used in the experiment was sterilized before use. For the preparation of the 1% chitosan solution 1 g of chitosan powder was dispersed in 100 mL of 1% acetic acid. For the preparation of the 3% chitosan solution 3 g of chitosan powder was dispersed in 100 mL of 1% acetic acid. For both concentrations, the dispersions were stirred overnight at room temperature using a magnetic stirrer to promote polymer chain relaxation and formation of a homogeneous solution. Before inclusion in the MTT assay, the prepared solutions were clarified to remove physical impurities and sterilized. Acetic acid and Octenisept^®^ solutions were taken directly from their commercial containers using sterile glass pipettes and transferred into sterile dropper bottles. Since the original MTA liquid component was supplied by the manufacturer in dropper form, no additional preparation was required. Prepared solutions were stored at 4 °C until use. The initial pH values of the prepared solutions were measured at room temperature before application using a calibrated digital pH meter to support the interpretation of the possible effects of the solutions on cellular response and recorded (Table 2).

Disc specimens were prepared with a diameter of 5 mm and a height of 2 mm. The discs were produced using a two-part male-female PTFE mould system to obtain standardized size and form.

The unmodified MTA mixture was prepared according to the manufacturer’s instructions, using 0.2 g of powder and three drops of manufacturer-supplied liquid. The volume of three drops was determined as 0.1 mL, and the biomodified groups were mixed with an equivalent liquid volume. MTA disc specimens were allowed to set for 24 h at 37 °C in a humid environment. After setting, 10 discs from each group were transferred into sterile 6-well culture plates. Surface sterilization was performed in a biosafety cabinet by applying ultraviolet light to each surface of the discs for 30 min (each side).

MTA discs were fully immersed in 2.36 mL serum-free DMEM and extracted for 24 h at 37 °C. After extraction, the obtained extracts were sterilized using a 0.22 µm pore-size filter. No extraction procedure was performed for the liquid specimens. In these groups, the direct effects of the solutions on the cells were evaluated. Before application, the liquid specimens were sterilized using a 0.22 µm filter. Before cell application, MTA extracts were prepared at 100%, 50%, and 25% concentrations. The 50% concentration was obtained by 1:2 dilution, and the 25% concentration by 1:4 dilution. In the solution groups, 100%, 50%, 25%, and 12.5% concentrations were prepared. The 12.5% concentration was obtained by 1:8 dilution. After the extracts or solutions were applied to the cells, the plates were incubated at 37 °C in a humidified incubator containing 5% CO_2_. Cytotoxicity assessments were performed after 24 h and 72 h. These time points were selected to assess early and prolonged exposure responses, respectively. Phase-contrast microscopic images were acquired to qualitatively document hPDLFs morphology after exposure. Images were obtained at 4× magnification. No quantitative morphometric analysis was performed on the microscopic images.

### 2.4. MTT Assay

Cell viability was evaluated using the MTT assay within the ISO 10993-5 framework. According to ISO 10993-5, cell viability below 70% of the negative control was considered indicative of cytotoxic potential. Before the assay, the application medium was removed from the cells. Then, 100 µL of serum-free medium containing 10% MTT solution was added to each well. The plates were incubated for 3 h at 37 °C in a CO_2_-free incubator while minimizing light exposure. After incubation, the MTT-containing medium was removed, and 100 µL of dimethyl sulfoxide (DMSO) was added to each well to dissolve the formazan crystals. The plates were shaken at 300 rpm for 10 min at room temperature to allow complete dissolution and uniform color development. Optical density (OD) was measured at 570 nm with a 690 nm reference wavelength using a microplate spectrophotometer. Absorbance values were corrected by subtracting the 690 nm reference reading from the 570 nm reading. Relative cell viability was calculated by normalizing the corrected absorbance of each experimental group to that of the negative control, which was set as 100%. The percentage of relative cell viability was calculated using the following formula:

Cell viability (%) = (corrected absorbance of the experimental group/corrected absorbance of the negative control) × 100

### 2.5. Statistical Analysis

Statistical analysis was performed using GraphPad Prism version 10.5.0 (GraphPad Software, Boston, MA, USA). No custom computer code was used for data analysis. Data were presented as mean ± standard deviation. Since the primary comparisons were performed within each concentration and exposure time, statistical analyses were conducted separately for each concentration-time condition. The Shapiro–Wilk test was used to assess the normality assumption. Homogeneity of variance was evaluated using Brown-Forsythe and Bartlett tests. When normality and homogeneity of variance assumptions were met, one-way analysis of variance followed by Tukey’s multiple comparison test was used. When homogeneity of variance was not met, Welch ANOVA followed by Dunnett’s T3 multiple comparison test was applied. When the normality assumption was not met, the Kruskal–Wallis test followed by Dunn’s multiple comparison test was used. The significance level was set at α = 0.05.

## 3. Results

Cell viability remained high in the negative control group throughout all evaluations, whereas a marked cytotoxic response was observed in the positive control group. The positive control was used only to validate the test system and was not included in the statistical comparisons. These findings confirmed that the MTT assay performed as expected and that the experimental model was able to distinguish cytotoxic responses. In accordance with ISO 10993-5, cell viability values below 70% of the negative control were considered cytotoxic.

### 3.1. Effect of Direct Solution Exposure on hPDLFs Cell Viability

The solution groups applied directly to the cells, without being mixed with MTA, showed clear concentration- and time-dependent effects on hPDLFs cell viability. The initial pH values of the solutions were 5.93, 3.65, 4.32 and 4.91 for Octenisept^®^, 1% acetic acid, 1% chitosan and 3% chitosan, respectively.

**Figure 1:**
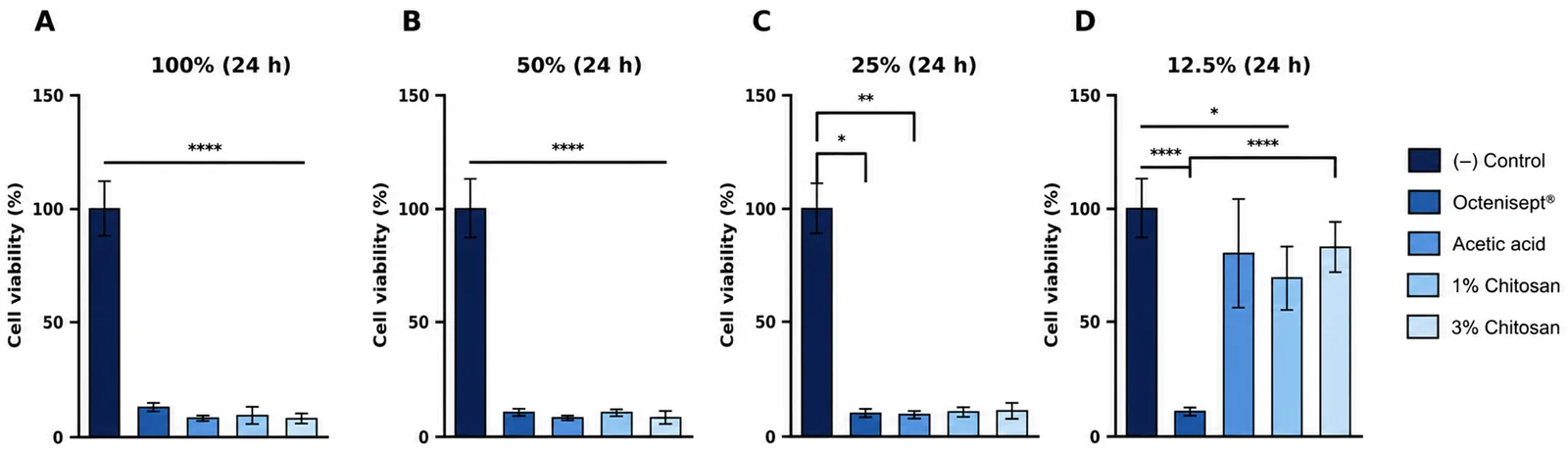
Cell viability of hPDLFs after 24 h direct exposure to the tested solutions. MTT-based cell viability percentages of hPDLFs exposed to Octenisept^®^, 1% acetic acid, 1% chitosan and 3% chitosan solutions at (**A**) 100%, (**B**) 50%, (**C**) 25% and (**D**) 12.5% concentrations for 24 h. Data are presented as mean ± standard deviation. The negative control was considered to have 100% cell viability. The 70% cell viability level was used as the cytotoxicity threshold according to ISO 10993-5. * *p* < 0.05, ** *p* < 0.01 and **** *p* < 0.0001 indicate statistically significant differences between the connected groups.

At 24 h, all solution groups showed a cytotoxic profile at 100%, 50% and 25% concentrations. At 100% and 50%, cell viability was markedly lower than that of the negative control in all groups, and all values remained below the 70% cytotoxicity threshold (*p* < 0.0001). At 25%, cell viability was significantly lower than the negative control only in the Octenisept^®^ (10.1%) and 1% acetic acid (9.5%) groups (*p* < 0.05 and *p* < 0.01, respectively); however, all groups remained below the 70% threshold at this concentration (Figure 1A–C). At 12.5%, a clearer separation among the solution groups was observed. Octenisept^®^ maintained its cytotoxic profile and was the only group with cell viability below the 70% threshold (10.7%). The 1% chitosan group showed a statistically significant difference compared with the negative control (*p* < 0.05); however, cell viability remained above the 70% threshold and was therefore not considered cytotoxic (Figure 1D).

**Figure 2:**
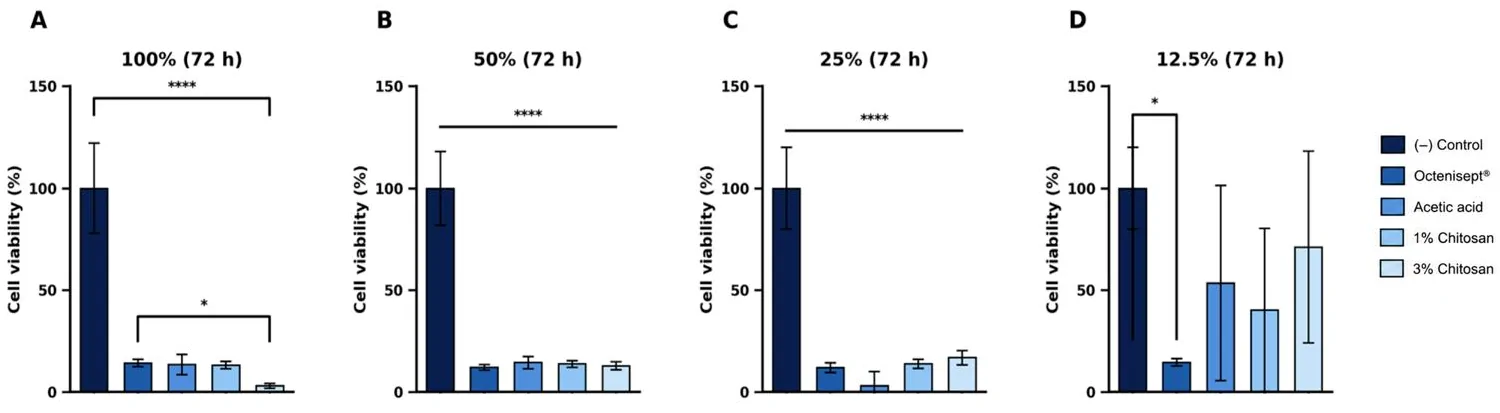
Cell viability of hPDLFs after 72 h direct exposure to the tested solutions. MTT-based cell viability percentages of hPDLFs exposed to Octenisept^®^, 1% acetic acid, 1% chitosan and 3% chitosan solutions at (**A**) 100%, (**B**) 50%, (**C**) 25% and (**D**) 12.5% concentrations for 72 h. Data are presented as mean ± standard deviation. The negative control was considered as 100% cell viability. The 70% cell viability level was used as the cytotoxicity threshold according to ISO 10993-5. * *p* < 0.05 and **** *p* < 0.0001 indicate statistically significant differences between the connected groups.

At 72 h, the cytotoxic response generally persisted in the direct solution exposure groups. At 100%, 50% and 25% concentrations, all solution groups remained below the 70% cytotoxicity threshold, and cell viability differed significantly from the negative control in all groups (*p* < 0.0001) (Figure 2A–C). At 100%, an additional significant difference was observed among the solution groups (*p* < 0.05) (Figure 2A). At 12.5%, Octenisept^®^ again showed a cytotoxic profile, with a cell viability of 14.5% and a significant difference from the negative control (*p* < 0.05). No statistically significant difference in cell viability was detected between the negative control and the other solution groups (*p* > 0.05). At this concentration, the acetic acid (53.5%) and 1% chitosan (40.3%) groups showed higher cell viability than Octenisept^®^, although their values remained below the 70% threshold. The 3% chitosan group showed comparatively higher cell viability (71.07%) and remained slightly above the cytotoxicity threshold (Figure 2D).

Under direct exposure conditions, Octenisept^®^ showed the most pronounced cytotoxic response among the tested solutions. In the acetic acid- and chitosan-containing solution groups, cytotoxicity decreased as the solution concentration was reduced; however, at higher concentrations, these solutions also markedly reduced hPDLFs cell viability. Representative phase-contrast microscopic images of hPDLFs after direct solution exposure are shown in Figure S2.

### 3.2. Effect of MTA-Containing Extract Groups on hPDLFs Cell Viability

The MTA-containing extract groups showed a more limited cytotoxic response than the direct solution exposure groups. The initial pH values of the extracts were 10.70, 11.01, 10.96, 11.11 and 10.98 for unmodified MTA, MTA + Octenisept^®^, MTA + 1% acetic acid, MTA + 1% chitosan and MTA + 3% chitosan, respectively.

**Figure 3:**
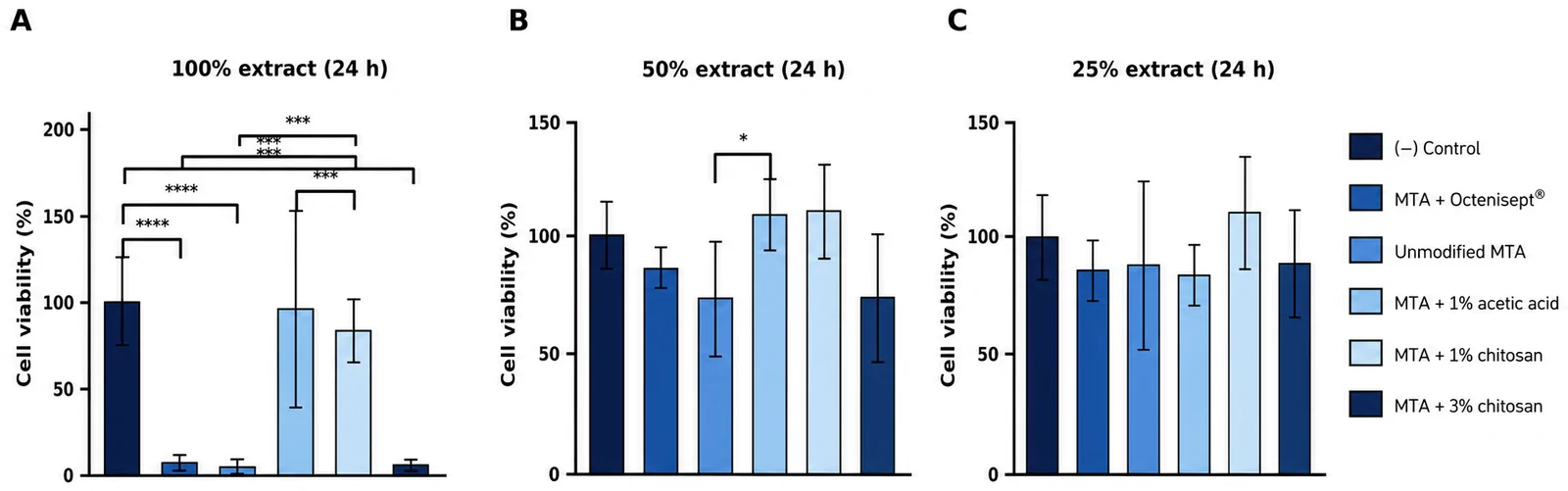
Cell viability of hPDLFs after 24 h exposure to MTA-containing extracts. MTT-based cell viability percentages of hPDLFs exposed to extracts of unmodified MTA, MTA + Octenisept^®^, MTA + 1% acetic acid, MTA + 1% chitosan and MTA + 3% chitosan at (**A**) 100%, (**B**) 50% and (**C**) 25% extract concentrations for 24 h. Data are presented as mean ± standard deviation. The negative control was considered to have 100% cell viability. The 70% cell viability level was used as the cytotoxicity threshold according to ISO 10993-5. * *p* < 0.05, *** *p* < 0.001 and **** *p* < 0.0001 indicate statistically significant differences between the connected groups.

At 24 h, cell viability remained above the 70% threshold in the MTA + 1% acetic acid and MTA + 1% chitosan groups at the 100% extract concentration. The MTA + 1% chitosan group (83.1%) differed significantly from unmodified MTA (5.008%), MTA + Octenisept^®^ (7.259%) and MTA + 3% chitosan (5.85%) (*p* < 0.001), whereas no significant difference was detected between MTA + 1% chitosan and MTA + 1% acetic acid (95.55%) (*p* > 0.05). Cell viability remained below the 70% threshold in the unmodified MTA, MTA + Octenisept^®^ and MTA + 3% chitosan groups. These groups also differed significantly from the negative control (*p* < 0.0001), while no significant difference was detected among them (*p* > 0.05). These findings indicate that, at the early time point and under undiluted extract conditions, Octenisept^®^ and 3% chitosan biomodifications did not provide a cell viability advantage over unmodified MTA (Figure 3A).

At the 50% extract concentration, cell viability remained above the 70% threshold in all MTA-containing groups. The MTA + 1% acetic acid (108.5%) and MTA + 1% chitosan (110.1%) groups showed comparatively higher cell viability values. Although cell viability was lower in the unmodified MTA (72.82%) and MTA + 3% chitosan (73.34%) groups, both remained above the cytotoxicity threshold. At this concentration, a statistically significant difference was detected only between the unmodified MTA and MTA + 1% acetic acid groups (*p* < 0.05), with higher cell viability in the MTA + 1% acetic acid group. No significant difference was detected between the negative control and the other biomodified MTA groups (*p* > 0.05) (Figure 3B).

At the 25% extract concentration, all groups showed a non-cytotoxic profile, and no statistically significant difference was detected among the groups (*p* > 0.05). However, the unmodified MTA group (73.85%) showed comparatively lower cell viability, whereas the MTA + 1% chitosan group (110.8%) showed comparatively higher cell viability (Figure 3C).

**Figure 4:**
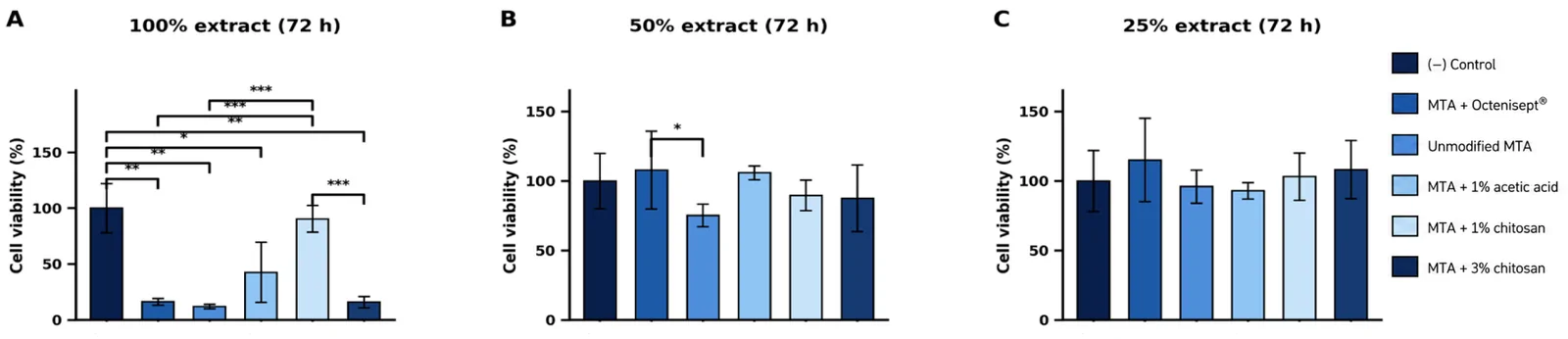
Cell viability of hPDLFs after 72 h exposure to MTA-containing extracts. MTT-based cell viability percentages of hPDLFs exposed to extracts of unmodified MTA, MTA + Octenisept^®^, MTA + 1% acetic acid, MTA + 1% chitosan and MTA + 3% chitosan at (**A**) 100%, (**B**) 50% and (**C**) 25% extract concentrations for 72 h. Data are presented as mean ± standard deviation. The negative control was considered as 100% cell viability. The 70% cell viability level was used as the cytotoxicity threshold according to ISO 10993-5. * *p* < 0.05, ** *p* < 0.01 and *** *p* < 0.001 indicate statistically significant differences between the connected groups.

At 72 h, the differences among the groups became more pronounced at the 100% extract concentration. Cell viability remained below the cytotoxicity threshold in the unmodified MTA (12.04%), MTA + Octenisept^®^ (16.29%), MTA + 1% acetic acid (42.73%) and MTA + 3% chitosan (16.07%) groups. Compared with the negative control, statistically significant differences were observed for MTA + Octenisept^®^ (*p* < 0.01), unmodified MTA (*p* < 0.01), MTA + 1% acetic acid (*p* < 0.05) and MTA + 3% chitosan (*p* < 0.01). In contrast, cell viability in the MTA + 1% chitosan group (90.41%) remained above the cytotoxicity threshold. This group differed significantly from all other MTA-containing extract groups (*p* < 0.001) (Figure 4A). This finding shows that MTA + 1% chitosan was the only biomodified MTA group that maintained cell viability above the cytotoxicity threshold under the 72 h, 100% extract condition, in which unmodified MTA and the other biomodified MTA groups showed cytotoxic profiles.

At the 50% extract concentration, cell viability remained above the cytotoxicity threshold in all groups. A statistically significant difference was detected only between the unmodified MTA (75.26%) and MTA + Octenisept^®^ (107.8%) groups (*p* < 0.05). The unmodified MTA group showed comparatively the lowest cell viability at this concentration and time point (Figure 4B). At the 25% extract concentration, cell viability remained above the cytotoxicity threshold in all groups, and no statistically significant difference was detected among the groups (*p* > 0.05) (Figure 4C).

Overall, MTA + 1% chitosan was the only biomodified MTA group that showed a non-cytotoxic profile at all extract concentrations and at both time points. Therefore, the null hypothesis was rejected. Under direct solution exposure, Octenisept^®^ showed the most pronounced cytotoxic profile. When the agents were incorporated into the MTA matrix, the cytotoxic response was generally reduced. The clearest separation among the MTA-containing groups was observed at 72 h under the 100% extract condition. Under this condition, unmodified MTA, MTA + 1% acetic acid, MTA + 3% chitosan and MTA + Octenisept^®^ showed cytotoxic profiles, whereas MTA + 1% chitosan maintained non-cytotoxic cell viability values. Representative phase-contrast microscopic images of hPDLFs after MTA extract exposure are shown in Figure S3.

## 4. Discussion

In this study, the MTT-based cytotoxicity profile of MTA modified using a bioinspired chitosan-based approach was evaluated in hPDLFs. Octenisept^®^ was used as an antiseptic comparator biomodifier, and 1% acetic acid was included as the vehicle control. The controlled design allowed the biomodified MTA extracts to be compared with unmodified MTA, the acetic acid vehicle control, and the corresponding direct solution exposure conditions. This design helped distinguish the effects of the MTA matrix, biomodifier type, vehicle system, extract concentration, and exposure time on the cellular response. The principal finding was that MTA + 1% chitosan maintained hPDLF viability above the ISO 10993-5 cytotoxicity threshold at all extract concentrations and at both 24 and 72 h. This difference was most evident after 72 h under undiluted extract conditions, where MTA + 1% chitosan showed higher cell viability than the other MTA-containing groups. Therefore, the null hypothesis was rejected.

The main contribution of this study is not the general observation that MTA-based materials affect cell viability, since this has already been reported in previous studies [8,9,10]. The contribution lies in the controlled comparison of different biomodification strategies within the same experimental model. By including unmodified MTA, an acetic acid vehicle control, two chitosan concentrations, Octenisept^®^-modified MTA, and the corresponding direct solution groups, the present design allowed for a more precise interpretation of whether the observed cellular response was associated with the MTA matrix, the biomodifying agent, the acidic vehicle, or the exposure condition. This distinction is relevant because a bioinspired material modification should be judged based on more than the origin or proposed function of the additive. The modified material should also preserve an acceptable biological response in a relevant cell model.

MTT analysis is widely used to assess cellular metabolic activity and relative cell viability in in vitro cytotoxicity studies [41,42]. In the present study, high cell viability in the negative control group and a marked cytotoxic response in the 10% DMSO positive control group supported the ability to distinguish biological differences in this experimental system. According to ISO 10993-5, cell viability below 70% of the negative control is considered indicative of cytotoxic potential [43], and this threshold was used for interpretation. However, MTT mainly reflects metabolic activity and does not directly measure cell death. Thus, a reduction in MTT absorbance should be interpreted as a reduction in relative cellular metabolic activity under the tested conditions, not as direct evidence of apoptosis, necrosis, or irreversible cell damage. MTT data may also be affected by the chemical environment of the material extracts, including pH, ion release, redox-active components, coloured components, particles, or possible interactions with the formazan reaction [44]. For this reason, the present findings should be regarded as preliminary MTT-based cytotoxicity screening data in hPDLFs. The use of negative and positive controls, the separate evaluation of solution and MTA extract groups, and the assessment of concentration- and time-dependent responses strengthen the interpretation of the results.

The use of hPDLFs is biologically relevant for evaluating endodontic repair materials. These materials may contact periodontal ligament and periradicular tissues during perforation repair, root-end procedures, or apical extrusion [8,37]. Periodontal ligament fibroblasts are involved in root-surrounding tissue homeostasis, extracellular matrix metabolism, wound healing, and regenerative responses [36]. Therefore, hPDLFs provide a clinically relevant cell model for evaluating the cellular response to MTA and biomodified MTA formulations [8,37]. The extract method used in this study is also one of the in vitro cytotoxicity approaches described in ISO 10993-5 [43]. Application of material extracts to hPDLFs allowed the effects of soluble components released from MTA-based materials to be assessed under standardised experimental conditions.

A clear difference was observed between the direct solution groups and the MTA extract groups. Under direct solution exposure, all tested solutions showed cytotoxic profiles at 100%, 50%, and 25% concentrations. Octenisept^®^ remained cytotoxic even at 12.5% after 24 and 72 h, indicating a marked cellular effect under direct exposure. When Octenisept^®^ was incorporated into the MTA matrix, cell viability remained above the 70% threshold at 50% and 25% extract concentrations, although cytotoxicity persisted under undiluted extract conditions at both time points. This finding suggests that incorporation into the MTA matrix may modulate the cellular effect of Octenisept^®^ by reducing direct exposure or altering the release and dilution behaviour of soluble components. This interpretation is consistent with the broader literature showing that additives or alternative mixing liquids can alter the physicochemical and biological behaviour of MTA-based materials [6,15,16,17]. However, direct evidence on the release behaviour of Octenisept^®^ from MTA is not available from the present study. The matrix-related modulation was also insufficient to eliminate cytotoxicity under undiluted extract conditions. Therefore, testing biomodifiers only as isolated solutions may not fully reflect their biological behaviour after incorporation into an MTA-based material.

Octenidine dihydrochloride is a broad-spectrum cationic antiseptic that can disrupt microbial membrane integrity through electrostatic interactions with negatively charged membrane components [18,19,20]. In the endodontic literature, octenidine has been reported to be effective against resistant microorganisms such as Enterococcus faecalis and Candida albicans [22,23]. The same cationic membrane interaction may also help explain the reduced viability observed in eukaryotic cells, particularly under direct exposure or high-concentration conditions. Coaguila-Llerena et al. reported that the effects of octenidine hydrochloride on L929 cells and human periodontal ligament cells should be considered carefully from a cellular safety perspective [25,39]. The present finding that Octenisept^®^ showed cytotoxicity at all concentrations under direct solution exposure is consistent with this concern. When Octenisept^®^ was incorporated into MTA, its cytotoxic effect was reduced in diluted extracts but remained evident under undiluted conditions. Therefore, Octenisept^®^ did not provide a cell viability advantage over unmodified MTA at the highest extract concentration.

The biological effect of Octenisept^®^ should also be interpreted in relation to the complete commercial formulation, not octenidine dihydrochloride alone. Octenisept^®^ contains 0.1% octenidine dihydrochloride and 2% phenoxyethanol. Stahl et al. reported that when using Octenisept^®^, 2-phenoxyethanol may have higher permeation potential than octenidine [45]. The cytotoxic response observed in the Octenisept^®^ groups may therefore reflect the effect of the commercial formulation as a whole. This point is important because the present study tested Octenisept^®^ as the commercial product, rather than isolated octenidine dihydrochloride.

Unmodified MTA showed a concentration- and time-dependent cytotoxicity profile. It was cytotoxic at the undiluted extract concentration after both 24 and 72 h, whereas cell viability increased above the cytotoxicity threshold at 50% and 25% dilutions. This finding agrees with previous studies reporting that the cellular response to calcium silicate-based materials varies according to extract concentration and exposure time [8,9,10]. Although MTA is generally regarded as biocompatible, reduced cell viability has been reported under high-concentration or early-stage extract conditions [8,9]. The present findings therefore support the view that the biological response to MTA is not fixed, but depends on concentration, exposure time, and material condition. In this context, the more favourable response of MTA + 1% chitosan compared with unmodified MTA at 72 h under undiluted extract conditions is an important comparative finding.

When the chitosan groups were considered, a clear concentration-dependent difference was observed between MTA extracts prepared with 1% and 3% chitosan. Chitosan is a natural, cationic, and biodegradable polysaccharide obtained by the deacetylation of chitin [31,34,40]. From a biomimetic perspective, chitosan is relevant because its polysaccharide backbone, cationic amino groups, and film- or scaffold-forming capacity are widely used in tissue engineering and regenerative biomaterials [27,31,34,40]. These properties make chitosan a bioinspired polymeric candidate for modifying calcium silicate-based repair materials. Its antimicrobial effect is mainly attributed to electrostatic interactions between protonated amino groups and negatively charged components on microbial cell surfaces [29,30,31]. However, the biological behaviour of chitosan can be affected by degree of deacetylation, concentration, solubility, viscosity, and pH [31,32,33]. These formulation-related factors are directly relevant to the present study, because the chitosan solutions were prepared in 1% acetic acid and then incorporated into the MTA matrix.

The most favourable response was observed in the MTA + 1% chitosan group. This group maintained cell viability above the 70% threshold at all extract concentrations and at both time points. Under the 72 h undiluted extract condition, unmodified MTA, MTA + 1% acetic acid, MTA + 3% chitosan, and MTA + Octenisept^®^ showed cytotoxic profiles, whereas MTA + 1% chitosan remained above the cytotoxicity threshold and differed significantly from the other MTA-containing extract groups. This finding indicates that, under the tested extract conditions, 1% chitosan produced the most favourable hPDLF viability response among the biomodified MTA formulations.

The acetic acid vehicle group was essential for interpreting the chitosan findings. MTA + 1% acetic acid was non-cytotoxic at the undiluted extract concentration after 24 h but became cytotoxic under the same concentration after 72 h. In contrast, MTA + 1% chitosan maintained a non-cytotoxic profile under the 72 h undiluted extract condition. This difference indicates that the favourable response in the MTA + 1% chitosan group cannot be attributed only to the acetic acid vehicle. Rather, incorporation of 1% chitosan into the MTA system was associated with preservation of hPDLF viability under a condition in which the vehicle control showed cytotoxicity. Since chitosan usually requires an acidic vehicle for dissolution [31,34], separate evaluation of the vehicle effect is necessary when interpreting chitosan-based MTA biomodifications.

MTA + 3% chitosan showed a different response. It was cytotoxic at the undiluted extract concentration after both 24 and 72 h, although cell viability rose above the cytotoxicity threshold at 50% and 25% dilutions. This finding indicates that the effect of chitosan in MTA biomodification is concentration-dependent. A higher chitosan concentration may provide stronger cationic interactions and greater antimicrobial potential, but the same property may also increase interactions with cell membranes and reduce cell viability. The present data should not be interpreted as showing that chitosan is intrinsically cytotoxic. A more appropriate interpretation is that 3% chitosan did not provide a cell viability response comparable to 1% chitosan under undiluted extract conditions. This interpretation is consistent with reports that chitosan-related biological responses depend on concentration, solubility, viscosity, and cationic charge density [46,47].

Several formulation-related mechanisms may explain why 1% chitosan showed a more favourable MTT-based response than 3% chitosan under undiluted extract conditions. First, increasing the chitosan concentration increases polymer content and may change viscosity, since chitosan solution behaviour is affected by concentration, solubility, and polymer characteristics [31,34]. In the present MTA system, these changes may influence powder-liquid wetting, ion diffusion, and early hydration of the calcium silicate matrix. The biological behaviour of MTA is closely related to hydration, calcium hydroxide formation, alkalinisation, and calcium ion release; therefore, changes in hydration kinetics or soluble component release may influence the cellular response [5,6]. Second, a higher chitosan concentration may increase the density of protonatable amino groups [29,30,31,34]. This cationic character contributes to antimicrobial activity through electrostatic interactions with negatively charged microbial surfaces [29,30]. However, a higher cationic charge density may also increase interactions with eukaryotic cell membranes under extract exposure conditions. In the present study, this may partly explain the lower MTT-based metabolic activity observed in the MTA + 3% chitosan group. Third, a more concentrated polymer phase within the MTA matrix may alter the release behaviour of alkaline or soluble components [6,15,16,17]. The similar initial pH values of the MTA extracts indicate that initial alkalinity alone does not explain the difference between the 1% and 3% chitosan groups. Thus, the more favourable response of MTA + 1% chitosan is more likely related to a balance among polymer concentration, matrix hydration, ion release, viscosity, and cell-extract interactions than to pH alone. These interpretations remain mechanistic hypotheses, because calcium ion release, hydration products, viscosity, and microstructural properties were not directly measured.

The absence of physicochemical characterization is an important limitation. Incorporation of chitosan, acetic acid, or Octenisept^®^ into MTA may affect hydration, setting time, calcium ion release, pH stability, solubility, mechanical properties, surface morphology, and microstructure. These properties are relevant to both biological behaviour and clinical handling. In the present study, only the initial pH values of the extracts and direct solutions were recorded to support biological interpretation. pH measurement alone is not sufficient to characterize the physicochemical consequences of biomodification. Future studies should therefore combine cytocompatibility testing with setting time analysis, calcium ion release, time-dependent pH monitoring, solubility, mechanical testing, surface morphology, and microstructural characterization.

The clinical meaning of the findings should be interpreted with caution. The extract concentrations used in this study represent standardized in vitro exposure conditions and do not reproduce the dynamic clinical environment. In vivo, material setting, tissue fluid exchange, buffering capacity, local dilution, blood contamination, immune response, and tissue repair processes may alter the concentration and biological effects of released components. Therefore, the present findings should not be taken as direct evidence of clinical safety or efficacy. They should be interpreted as controlled preclinical screening data identifying MTA + 1% chitosan as a candidate formulation that requires further investigation.

The rationale for MTA biomodification is to improve antimicrobial performance while preserving cytocompatibility. Future studies should therefore assess biological safety and functional efficacy together. Antimicrobial testing against endodontic microorganisms, antibiofilm analyses, physicochemical characterization, calcium ion release, setting properties, mechanical performance, and longer-term biological assessment are needed. Studies using complementary cell models, three-dimensional culture systems, and dentin models would also provide more clinically relevant data on the translational potential of chitosan-modified MTA formulations.

To the best of our knowledge, this is one of the first studies to evaluate MTA mixed with Octenisept^®^ and MTA mixed with 1% acetic acid as a separate vehicle control in parallel with chitosan-modified MTA extracts and corresponding direct solution exposure groups. The most distinct separation among the MTA-containing groups was observed after 72 h under undiluted extract conditions. These findings support further investigation of 1% chitosan-modified MTA as a candidate formulation for biological, antimicrobial, and physicochemical evaluation.

## 5. Conclusions

Within the limitations of this MTT-based in vitro screening model, MTA + 1% chitosan showed the most favourable hPDLF viability profile among the tested biomodified MTA formulations. Direct exposure to the tested solutions, particularly Octenisept^®^, produced stronger cytotoxic effects than the corresponding MTA extracts, indicating that incorporation into the MTA matrix modulated the cellular response to the biomodifying agents. The time-dependent effect of the acetic acid vehicle supports its inclusion as a separate control in chitosan-based MTA biomodification studies. The controlled design provides preliminary cytotoxicity data identifying 1% chitosan-modified MTA as a candidate formulation for further biological, antimicrobial, and physicochemical investigation. These findings should not be directly extrapolated to clinical practice. Before clinical relevance or translational potential can be established, complementary biological assays, antimicrobial and antibiofilm analyses, calcium ion release testing, time-dependent pH evaluation, setting time, solubility, mechanical testing, surface morphology, and microstructural characterization are required.

## Figures and Tables

**Table 1:** Tested materials, suppliers and composition details.

Material/Solution	Supplier	Composition/Description	Catalogue/Lot No.
Biofactor MTA	Imicryl Dental, Konya, Turkey	Powder. Tricalcium silicate, dicalcium silicate, tricalcium aluminate, ytterbium oxide.	Cat. No. BFC01000; Lot No. P928
		Liquid. Gel containing water-soluble carboxylated polymer & demineralized water.	
Acetic acid 1%	Izmir Teknik Kimya, İzmir, Turkey	Liquid.	Cat. No. İTK.A27.1002; Lot No. not available
Chitosan CP200–300	ZAG Kimya, İstanbul, Turkey	off-white powder.	Cat. No. ZK.100522.0050; Lot No. Z2025120107
Octenisept^®^	Schülke & Mayr GmbH, Norderstedt, Germany	Liquid. 0.1% octenidine dihydrochloride. 2% phenoxyethanol.	Cat. No. 121464; Lot No. 1561156

**Table 2:** Initial pH values of tested MTA extracts & direct solutions.

Tested Material/Solution	Exposure Type	Initial pH Value
Unmodified MTA mixture	MTA extract	10.70
MTA powder + Octenisept^®^	MTA extract	11.01
MTA powder + 1% acetic acid	MTA extract	10.96
MTA powder + 1% chitosan in 1% acetic acid	MTA extract	11.11
MTA powder + 3% chitosan in 1% acetic acid	MTA extract	10.98
1% acetic acid	Direct solution	3.65
1% chitosan in 1% acetic acid	Direct solution	4.32
3% chitosan in 1% acetic acid	Direct solution	4.91
Octenisept^®^	Direct solution	5.93

## Data Availability

The data supporting the findings of this study are included within the article. Additional raw data generated and analyzed during the current study are not deposited in a public repository and will be made available by the corresponding author on reasonable request. No genomic, transcriptomic, sequence, clinical participant-level, or identifiable personal data were generated in this study.

## Supplementary Materials

The following supporting information can be downloaded at: https://www.mdpi.com/article/10.3390/biomimetics11080528/s1, Figure S1. Experimental design and grouping scheme; Figure S2. Representative phase-contrast microscopic images after direct solution exposure; Figure S3. Representative phase-contrast microscopic images after MTA extract exposure; Supplementary Image Archive: Original phase-contrast microscopic images.

## Abbreviations

The following abbreviations are used in this manuscript:

MTA	Mineral trioxide aggregate
hPDLFs	human periodontal ligament fibroblasts
NaOCl	sodium hypochlorite
CHX	chlorhexidine gluconate
DMEM	Dulbecco’s modified Eagle’s medium
FBS	fetal bovine serum
PBS	phosphate-buffered saline
DMSO	dimethyl sulfoxide
OD	Optical density
